# Predictors of Cytoreductive Nephrectomy for Metastatic Kidney Cancer in SEER and Metropolitan Detroit Databases

**DOI:** 10.15586/jkcvhl.2019.121

**Published:** 2019-10-28

**Authors:** Ulka Vaishampayan, Julie George, Fawn Vigneau

**Affiliations:** Department of Oncology, Karmanos Cancer Center, Wayne State University, Detroit, MI, USA

**Keywords:** comorbidities, kidney cancer, nephrectomy, prognostic factors, SEER registry

## Abstract

Patients without cytoreductive nephrectomy (CN) are inadequately represented in metastatic renal cell carcinoma (RCC) clinical trials. The characteristics that impact the decision of CN were explored in the SEER database. Data on primary, regional, or distant (metastatic) stage kidney cancer over the period 2000–2013 were extracted from the National Cancer Institute Surveillance, Epidemiology, and End Results Program (SEER-18) database. A sub-analysis of Metropolitan Detroit cases, to evaluate the influence of comorbidities, was conducted. Logistic regression was used to calculate the odds ratios, and Cox model was used to calculate hazard ratios; 37% of 21,052 metastatic RCC cases had CN performed. CN demonstrated significant survival advantage (HR = 0.31, 95% confidence interval [CI]: 0.30–0.33). Comorbidity data were available on 76% of distant RCC cases from the Detroit SEER database. Neither hypertension, diabetes mellitus nor the number of comorbidities (0, 1 or 2) had a statistically significant impact on the likelihood of CN. Majority of patients (63%) with distant-stage RCC do not undergo CN and have a median overall survival (OS) of 3 months as compared to a median OS of 18 months for patients who have undergone CN. Patient demographics and tumor characteristics make a significant impact on the incidence of CN. The impact of comorbidities (number and type) was modest and not statistically significant. The optimal management of patients with synchronous primary and metastatic RCC needs to be prospectively evaluated in the setting of contemporary systemic therapy.

## Introduction

Cytoreductive nephrectomy (CN) in the presence of metastatic disease is a unique feature of the therapeutics of advanced renal cancer (RCC). There are two randomized trials demonstrating the overall survival (OS) benefit favoring CN in metastatic disease compared to systemic interferon therapy alone (1, 2). The only systemic therapies available previously were cytokine-based regimens such as interleukin and interferon during the time period when these CN trials were conducted. Within the last decade, 10 additional therapies have attained Food and Drug Administration (FDA) approval in metastatic renal cancer (3). Until recently, no prospective study had been completed evaluating the role of CN in the setting of contemporary systemic therapies. The results of two randomized trials comparing CN followed by sunitinib versus systemic therapy with sunitinib followed by CN [SURTIME trial] and nephrectomy followed by sunitinib versus sunitinib alone (CARMENA) were recently reported. Both studies indicated that the clinical outcomes with sunitinib alone were at least similar and possibly slightly improved in the metastatic RCC patients randomized to deferred or no CN (4, 5). The studies showed that CN does not impart additional survival benefit in the setting of effective systemic therapy, and a proportion of patients are deprived of systemic therapy due to complications of CN. In summary, the concept of CN being an essential component of metastatic RCC therapy is being seriously questioned. These data need to be considered in future therapeutic decisions in metastatic RCC.

The National Cancer Database (NCDB), which reflects community-based data from hospitals with Commission on Cancer accreditation, continues to reveal that the incidence of CN in distant-/advanced-stage RCC is seen in about a third of the patients (6). Unlike clinical trials of systemic therapy, where 80–100% of patients are receiving nephrectomy, the majority of the patients in the real-world treatment of RCC are not receiving CN as primary management. The overall survival outcome has improved in advanced RCC; however, it also appears that the patients with CN received the maximum benefit (7). Despite randomized evidence to the contrary, the OS results in SEER show that CN patients have a large magnitude of benefit as compared to non-CN patients. This finding may be confounded by the fact that the patients undergoing CN are probably younger, healthier, and less likely to have aggressive or poor risk disease, factors that portend an improved prognosis regardless of nephrectomy status. In an attempt to overcome the confounding effect of conflicting prognostic factors, we analyzed and reported on a clinical trial population with metastatic disease and found that CN maintained a significant positive impact on clinical outcomes (8).

In the targeted therapy era, we conducted a SEER data analysis of outcomes in patients who have undergone CN versus those who have not undergone CN. The results revealed that only the post-nephrectomy population appeared to benefit from advances in systemic therapies (8). Clearly, an intervention that would increase the possibility of CN in metastatic RCC would likely improve outcomes. The controversy of whether CN should be standard in metastatic RCC continues, with the recent randomized prospective trials suggesting minimal contribution of CN in improving outcomes in metastatic RCC. In addition, there is an added nuance of analyzing the role of CN in the presence of contemporary immune checkpoint combination therapy. There is preclinical evidence that suggests that the immune checkpoint inhibitors would be primed to demonstrate improved efficacy in the presence of the primary tumor. Randomized trials of systemic therapy with immune checkpoint inhibition with or without CN are being planned in this setting.

We attempted to evaluate the magnitude of the problem by assessing the incidence of CN in metastatic RCC within the SEER data. We analyzed the factors that are likely to impact the decision of CN in the initial management of advanced RCC. We explored demographic and disease-related factors. In addition, we assessed the impact of comorbidities in receipt of initial CN using additional data from the Metropolitan Detroit Cancer Surveillance System (MDCSS). We conducted an analysis of distant/metastatic cases of RCC within the SEER-18 data between 2000 and 2013. The intention was to evaluate the incidence of CN in the contemporary systemic therapy era and to assess the factors that impacted the decision of CN. We chose to explore the impact of a few key comorbidities such as renal function, hypertension, diabetes mellitus, and lung disease.

## Methods

**Figure 1:**
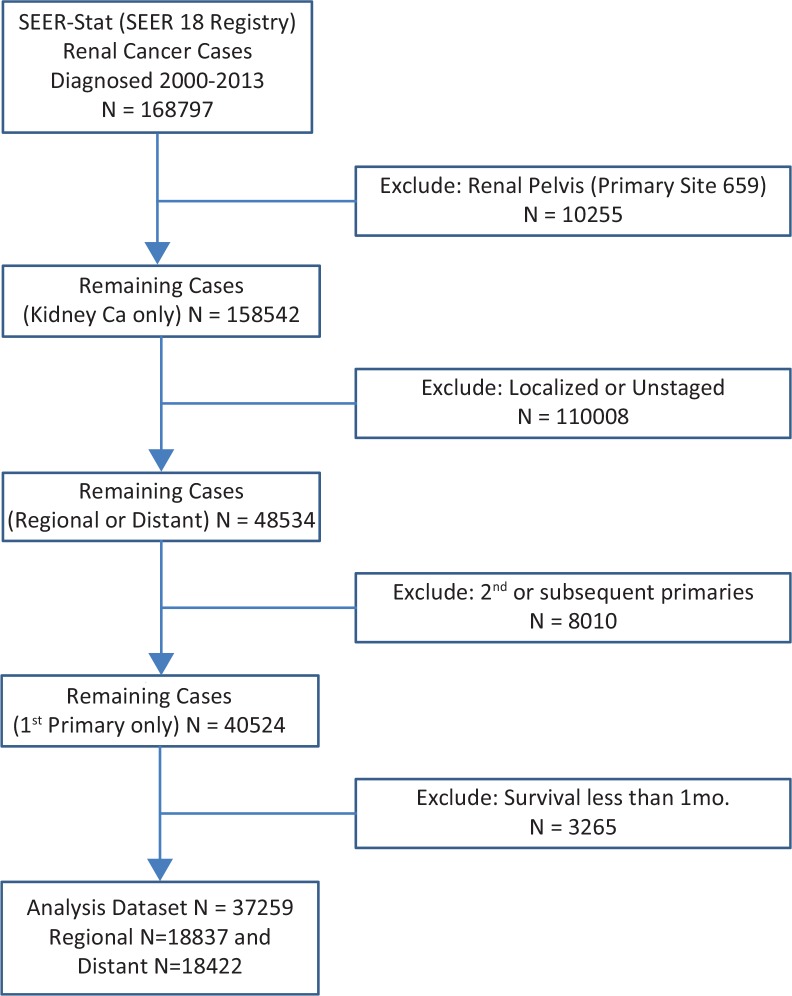
Consort diagram of patient population included in analyses.

The National Cancer Institute Surveillance, Epidemiology, and End Results Program (SEER-18) contains regional research data (1973–2011) submitted in November 2013 with the Katrina/Rita population adjustment, collected from 18 American cancer registries, chosen for their data quality and population diversity. The MDCSS is a data contributor to the SEER Program. Data for the primary analyses were extracted using SEER*Stat software (version 8.3.1), for patients diagnosed with renal cancer between the years 2000 and 2013. We restricted the analysis to the first diagnosis of regional or distant stage kidney cancer, overall referred to as “metastatic” kidney cancer. Site-specific surgery codes 30, 40, 50, 70, or 80 were defined as having received nephrectomy. The main focus was advanced or metastatic cases with RCC which are coded as “distant” in SEER. Logistic regression analysis was used to calculate odds ratios (OR) of a patient who has undergone nephrectomy, adjusted for age at diagnosis, race, gender, marital status, insurance and disease histology, and grade. Cox proportional hazards regression analysis was used to calculate adjusted hazard ratios (HR) to estimate the overall risk of death. Hazard ratios were adjusted for nephrectomy as well as for all other variables listed above. Analyses were conducted using SAS software version 9.4 (SAS Institute Inc., Cary, NC). Patients with OS < 1 month were excluded from the analysis with the assumption that any treatment would not have a chance to impact their outcomes. Figure 1 depicts the flowchart of the patient population considered and selected for analysis.

We also performed a separate analysis using the same criteria for MDCSS data, extracted from the local SEER Database Management System (SEER*DMS) using SAS© v 9.4, to evaluate the influence of comorbidities, as these data are not collected by SEER. Logistic regression was used to calculate the odds ratios for receiving CN. Hazard ratios were calculated using Cox proportional hazards regression.

## Results

### Overview and predictors of nephrectomy

After exclusion of renal pelvis cases, final SEER-18 data for 2000–2013 had N = 40,524 patients. Case distribution by stage included regional stage (N = 19,472, 48%, 93% had nephrectomy) and distant stage (N = 21,052, 52%, 37% had nephrectomy). Our analyses were restricted to distant or metastatic stage patients only (Figure 1). The population characteristics of all SEER registry “regional” and “distant cases” are depicted in Table 1.

**Table 1:** Population Characteristics and Nephrectomy Rates of All SEER Kidney Cancer, Regional or Distant Stage: 2000–2013.

	Regional 18,837			Distant 18,422		
	N	Distribution (%)	Nephr Rate (%)	N	Distribution (%)	Nephr Rate (%)
**Registry**						
Alaska	22	0	91	28	0	32
Atlanta	540	3	92	558	3	41
California (excl SF,SJM, & LA)	4196	22	94	4195	23	43
Connecticut	869	5	93	794	4	37
Detroit	939	5	93	947	5	39
Georgia (greater)	1381	7	92	1307	7	39
Hawaii	356	2	98	276	1	45
Iowa	896	5	96	921	5	43
Kentucky	1234	7	94	1184	6	43
Los Angeles	1845	10	95	1830	10	45
Louisiana	1370	7	91	1334	7	38
New Jersey	1985	11	94	1849	10	41
New Mexico	538	3	91	522	3	33
Rural Georgia	32	0	97	37	0	35
San Francisco	779	4	93	848	5	45
San Jose	421	2	95	448	2	43
Seattle	977	5	94	980	5	42
Utah	457	2	95	364	2	50
**Age at diagnosis (year)**						
Under 20	626	3	98	502	3	91
20–39	577	3	96	452	2	55
40–49	1937	10	97	1855	10	56
50–59	4422	23	97	4511	24	51
60–69	5369	29	95	5184	28	42
70–79	4122	22	93	3762	20	31
80 or older	1784	9	78	2156	12	12
**Sex**						
Female	6280	33	92	6185	34	39
Male	12,557	67	95	12,237	66	43
**Race**						
Black	1612	9	90	1926	10	35
White	16,020	85	94	15,241	83	42
Other	1102	6	96	1208	7	44
Unknown	103	1	98	47	0	40
**Marital status**						
Married	11,556	61	96	10,482	57	46
Divorced	1700	9	92	1994	11	39
Single	2959	16	95	3077	17	45
Widowed	1890	10	85	2230	12	22
Unknown	732	4	85	639	3	33
**Insurance**						
Insured	8449	45	95	7573	41	43
Uninsured/medicaid	1530	8	93	1887	10	36
Unknown/missing	8858	47	93	8962	49	41
**Histology**						
Clear cell	14,287	76	94	13,952	76	39
Renal cell carcinoma, Chromophobe	662	4	98	125	1	76
Renal cell carcinoma, Sarcomatoid	488	3	95	878	5	62
Collecting duct carcinoma	90	0	94	98	1	67
Papillary carcinoma, Oxyphilic cell	0	0	-	1	0	100
Other non-clear cell	3310	18	93	3368	18	46
**Grade**						
Well differentiated	859	5	97	318	2	45
Moderately differentiated	5513	29	99	1750	9	71
Poorly differentiated	5902	31	98	3653	20	73
Undifferentiated	2319	12	98	2233	12	86
Unknown	4244	23	78	10,468	57	16
**Surgery**						
Nephrectomy	17,665	94		7660	42	
Other surgery	142	1		179	1	
No urgery	1016	5		10,500	57	
Unknown if Surgery done	14	0		83	0	

Nephr Rate, nephrectomy rate.

Adjusted predictors of statistically increased likelihood of receiving nephrectomy were age (≤63 years), male sex (white race), and marital status. An interaction effect was noted between gender and marital status with married males having a significantly higher nephrectomy rate (42%) than divorced, single, and widowed men (35, 38, and 18% respectively). Single males were significantly less likely to receive nephrectomy than females (OR 0.53, 95% confidence interval [CI] 0.44–0.64). Single females had the highest (44%) likelihood of nephrectomy. Non-clear cell histology and poorly differentiated grade had a higher possibility of receiving CN as compared to clear cell histology and well-differentiated cancer, respectively (Table 2).

**Table 2:** All SEER Distant Stage Kidney Cancer: 2000–2013; Odd Ratios for Likelihood of Nephrectomy.

Total	N 18422	Nephr. Rate 42 (%)	Unadjusted OR	Adjusted OR	95% CI	*p-value*
**Age at Diagnosis Diagnosis *(median 63)***						
(ref) Under 63	8982	53	1.00	1.00		
63 or Older	9440	31	0.39	0.41	(0.38–0.44)	*<0.001*
**Race *(47 missing)***						
(ref) Black	1926	35	1.00	1.00		
White	15241	42	1.34	1.51	(1.33–1.72)	*<0.001*
Other	1208	44	1.43	1.52	(1.25–1.84)	*<0.001*
Unknown	47	40		N/A		
**Insurance**						
(ref) Insured	7573	43	1.00	1.00		
Medicaid or Uninsured	1887	36	0.75	0.65	(0.57–0.75)	*<0.001*
Unknown or Missing	8962	41	0.93	1.17	(1.08–1.27)	*<0.001*
**Histology**						
(ref) Clear Cell	13952	39	1.00	1.00		
Non-Clear Cell	4470	50	1.59	1.58	(1.45–1.73)	*<0.001*
**Grade**						
(ref) Well differentiated	318	45	1.00	1.00		
Moderately differentiated	1750	71	3.06	3.35	(2.59–4.33)	*<0 001*
Poorly differentiated	3653	73	3.32	3.24	(2.53–4.13)	*<0 001*
Undifferentiated	2233	86	7.54	7.15	(5.49–9.31)	*<0 001*
Unknown	10468	16	0.24	0.23	(0.18–0.29)	*<0 001*
**Sex**						
(ref) Female	6185	39	1.00	*See interaction below*		
Male	12237	43	1.16			
**Marital Status**						
(ref) Married	10482	46	1.00	*See interaction below*		
Divorced	1994	39	0.75			
Single	3077	45	0.96			
Widowed	2230	22	0.34			
Unknown	639	33	0.58			
**Sex by Marital Status**						
Male	7767	46	1.00	1.03	(0.92–1.16)	*0.567*
Divorced						
(ref) Female	742	40	1.00	1.00		
Male	1252	38	0.93	0.79	(0.62–1.00)	*0.047*
Single						
(ref) Female	984	49	1.00	1.00		
Male	2093	43	0.76	0.53	(0.44–0.64)	*<0.001*
Widowed						
(ref) Female	1528	22	1.00	1.00		
Male	702	23	1.03	1.03	(0.79–1.34)	*0.963*
Unknown						
(ref) Female	216	28	1.00	1.00		
Male	423	35	1.43	1.33	(0.84–2.09)	*0.298*
Married						
(ref) Female	2715	46	1.00	1.00		

Italic values represent missing data.

### Survival status

**Figure 2:**
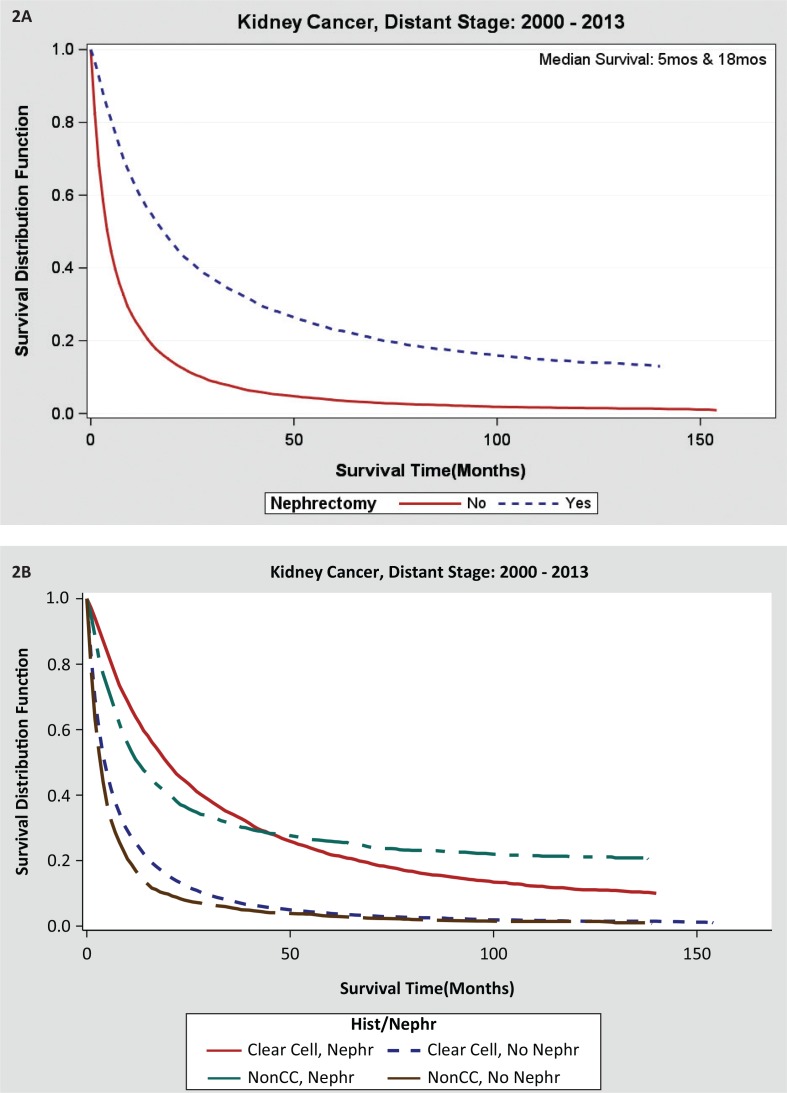
(A) Overall survival data for nephrectomy or non-nephrectomy patients with advanced kidney cancer in SEER database. (B) Overall survival outcomes in metastatic RCC by clear and non-clear histology with or without nephrectomy.

Receipt of nephrectomy demonstrated significant survival advantage (HR = 0.31, 95% CI: 0.30–0.33) in the adjusted hazards ratio model. Median OS was 3 months in the non-CN group and 18 months in the CN group (Table 3 and Figure 2A). The demographic predictors of improved survival were age <63, white race, married status, and insured status (Table 3). Clear cell histology (HR = 1.20, 95% CI: 1.16–1.24) and well-differentiated grade are the disease-related characteristics that portend for improved OS (Figure 2B). The latter demonstrated improved OS despite a lower, 43% CN rate as compared to 84% in the undifferentiated histology group. The histology grades in SEER of well, moderate, poor, and undifferentiated RCC are likely to be concordant with the conventional Fuhrman 1–4 grading (Table 3).

**Table 3:** All SEER Distant Stage Kidney Cancer (2000–2013) Survival Analysis (Median Survival, Adjusted Hazard Ratio, 95% Confidence Interval, and P-value).

Total	N 18422	Median Survival *(months)*		HR	95% CI	*p-value*
		Nephr. 18	No Nephr. 5			
**Age at Diagnosis *(median 63)***						
(ref) Under 63	8982	20	25	1.00		
63 or Older	9440	16	4	1.16	(1.12–1.20)	*<0.001*
**Sex**						
(ref) Female	6185	18	4	1.00		
Male	12237	19	5	0.99	(0.96–1.03)	*0.702*
**Race *(47 missing)***						
(ref) Black	1926	16	4	1.00		
White	15241	25	5	0.98	(0.93–1.02)	*0.342*
Other	1208	18	5	0.88	(0.81–0.95)	*0.002*
**Marital Status**						
(ref) Married	10482	17	5	1.00		
Divorced	1994	16	5	1.05	(1.00–1.11)	*0.051*
Single	3077	29	5	0.86	(0.82–0.90)	*<0.001*
Widowed	2230	14	4	1.09	(1.04–1.15)	*0.001*
Unknown	639	20	5	0.86	(0.79–0.94)	*0.001*
**Insurance**						
(ref) Insured	7573	20	5	1.00		
Medicaid or Uninsured	1887	21	5	1.03	(0.97–1.09)	*0.427*
Unknown or Missing	8962	16	4	1.16	(1.12–1.20)	*0.001*
**Histology**						
(ref) Clear Cell	13952	20	5	1.00		
Non-Clear Cell	4470	13	4	1.16	(1.12–1.21)	*<0.001*
**Grade**						
(ref) Well differentiated	318	35	9	1.00		
Moderately differentiated	1750	30	6	1.30	(1.13–1.49)	*<0.001*
Poorly differentiated	3653	18	4	1.86	(1.63–2.13)	*<0.001*
Undifferentiated	2233	11	3	2.47	(2.16–2.83)	*<0.001*
Unknown	10468	26	5	1.48	(1.30–1.69)	*<0.001*
**Surgery**						
No Nephrectomy	10762	-	5	1.00		
Nephrectomy	7660	18	-	0.34	(0.32–0.35)	*<0.001*

95% CI, 95% confidence interval; HR, hazard ratio; Nephr, nephrectomy.

### Comorbidity analyses in Metro Detroit Registry

**Figure 3:**
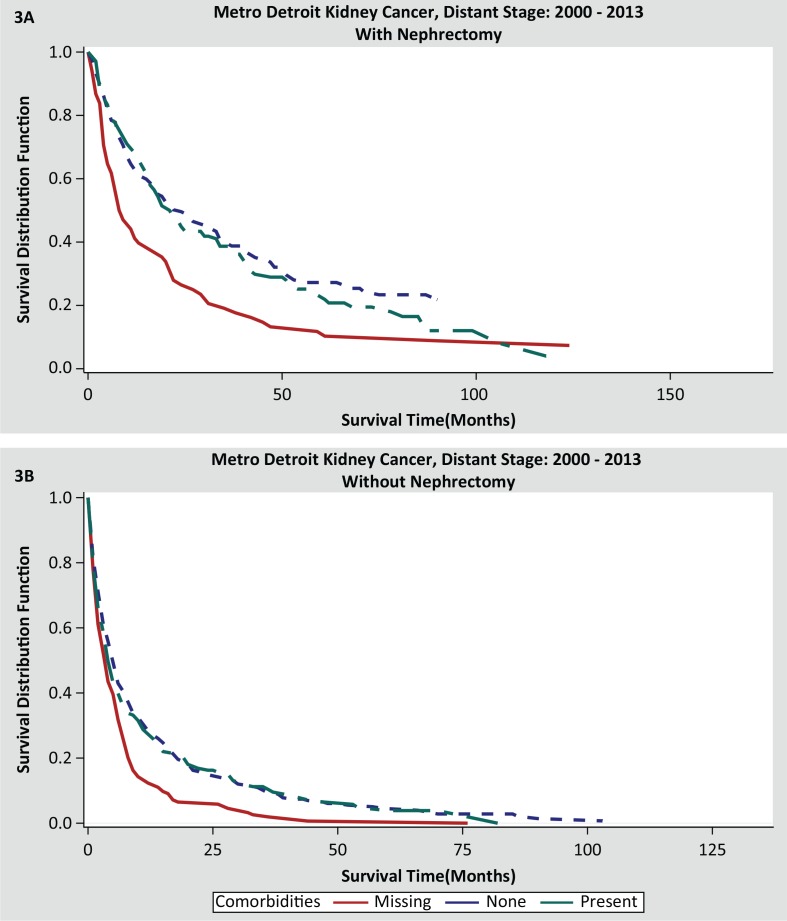
(A) Overall survival noted in cases with nephrectomy correlated with missing, absence, and presence of comorbidities. (B) Overall survival seen in cases with no nephrectomy correlated with missing, absence, and presence of comorbidities.

The data regarding comorbid conditions were only available for the Metro Detroit (MDCSS) Registry. While not collected as a population-based variable, comorbidity status was coded for 82% of regional and 76% of metastatic kidney cancers seen in Metropolitan Detroit. Nephrectomy was performed in 92% of regional stage and 34% of distant stage patients; our analysis pertains to only the distant-stage patients. Dichotomous comorbidities (yes or no for: hypertension, cardiac disease, diabetes mellitus, kidney disease, and lung disease) and number of comorbid conditions (none, 1, 2, 3+, and missing) are included in the patient characteristics of MDSCC (Table 4). The demographic and tumor characteristics were concordant with the all SEER data; 44.9% of the patients had none of the comorbidities. Adjusted demographic predictors of increased likelihood of receiving nephrectomy for Metropolitan Detroit patients were age < 64 years, non-black race, and married status. Non-clear cell histology (OR = 2.16, 95% CI: 1.42–3.28) and undifferentiated or high also predicted a higher likelihood of CN. Adding comorbidities and number of comorbidities to the model did not impact the likelihood of receiving nephrectomy. All comorbidity types and numbers were not statistically significant in terms of predicting the likelihood of CN and OS (Table 5). Patients with hypertension (OR = 1.75, 95% CI: 0.63–4.87) or diabetes mellitus had greater likelihood of receiving nephrectomy (OR = 1.16, 95% CI: 0.42, 3.21). The group of patients with missing comorbidity data (261/1104, 23.6%) had a significantly shorter OS in both CN and non-CN categories. The actual correlation of comorbidities with likelihood of CN remains elusive due to the group with missing data constituting 24% of the population; 26% of this group had a nephrectomy and demonstrated a median OS of 8 months as compared to the 76% with a median OS of 2 months (Tables 4 and 5; Figure 3A and 3B).

**Table 4:** Metropolitan Detroit Distant Stage Kidney Cancer: 2000–2013 Odd Ratios for Likelihood of Nephrectomy.

Total	N 1008	Distribution	Nephr. Rate 37 (%)	Adjusted OR	95% CI	*p-value*
**Age at Diagnosis *(median 64)***						
(ref) Under 64	498	49	51	1.00		
64 or Older	510	51	23	0.29	(0.21–0.39)	*<0.001*
**Sex**						
(ref) Female	338	34	36	1.00		
Male	670	66	37	0.87	(0.64–1.19)	*0.389*
**Race**						
(ref) Black	206	20	34	1.00		
White	788	78	38	1.48	(1.03–2.13)	*0.035*
Other	14	1	36	-		
**Marital Status**						
(ref) Married	507	50	40	1.00		
Divorced	118	12	31	0.60	(0.38–0.95)	*0.031*
Single	202	20	43	0.81	(0.56–1.19)	*0.285*
Widowed	146	14	21	0.51	(0.31–0.83)	*0.006*
Unknown	35	3	37	1.13	(0.52–2.44)	*0.757*
**Insurance**						
(ref) Insured	663	66	38	1.00		
Medicaid or Uninsured	277	27	40	1.12	(0.79–1.59)	*0.524*
Unknown or Missing	67	7	18	0.41	(0.21–0.81)	*0.010*
**Histology**						
(ref) Clear Cell	695	69	34	1.00		
Non-Clear Cell	313	31	45	1.68	(1.25–2.27)	*0.001*
**Grade**						
(ref) Well or Mod Diff	75	7	44	*left out of model because of the large proportion of unknowns*		
Poorly Diff	168	17	70			
Undifferentiated	152	15	81			
Unknown	613	61	16			
**Number of Comorbid conditions**						
(ref) None of the above	465	46	36	1.00		
One	165	16	41	1.25	(0.84–1.87)	*0.269*
Two or more	156	15	46	1.76	(1.17–2.66)	*0.007*
Missing data	222	22	31	0.80	(0.54–1.18)	*0.249*

95% CI, 95% confidence interval; Mod Diff, moderately differentiated.

**Table 5:** Metropolitan Detroit Distant Stage Kidney Cancer: 2000–2013 Survival Analysis by Nephrectomy Status for Demographic Characteristics and Comorbidities.

Total	N 1008	Median Survival *(months)*		HR	95% CI	*p-value*
		Nephr. 19	No Nephr. 5			
**Age at Diagnosis *(median 64)***						
(ref) Under 64	551	22	5	1.00		
64 or Older	553	15	5	1.08	(0.93–1.25)	*0.335*
**Sex**						
(ref) Female	370	19		1.00		
Male	734	20		1.00	(0.86–1.16)	*0.990*
**Race *(55 missing)***						
(ref) Black	232	30	4	1.00		
White	857	18	5	1.02	(0.86–1.21)	*0.824*
Other	15	-	-	-		
**Marital Status**						
(ref) Married	552	18	5	1.00		
Divorced	122	12	4	1.18	(0.95–1.46)	*0.145*
Single	222	43	4	0.76	(0.62–0.93)	*0.007*
Widowed	169	17	4	1.02	(0.82–1.25)	*0.891*
Unknown	39	16	8	0.71	(0.49–1.02)	*0.064*
**Insurance**						
(ref) Insured	720	18	5	1.00		
Medicaid or Uninsured	309	22	4	1.02	(0.86–1.22)	*0.784*
Unknown or Missing	75	31	4	1.35	(1.03–1.78)	*0.032*
**Histology**						
(ref) Clear Cell	755	20	5	1.00		
Non-Clear Cell	349	18.5	4	1.17	(1.01–1.36)	*0.037*
**Grade**						
(ref) Well or Mod Diff	76	35	9	*left out of model because of the large proportion of unknowns*		
Poorly Diff	178	19	4			
Undifferentiated	158	12	3			
Unknown	692	36	5			
**Surgery**						
No Nephrectomy	726	-	3	1.00		
Nephrectomy	378	19	-	0.40	(0.34–0.46)	*<0.001*
**Number of Comorbid conditions**						
(ref) None of the above	496	24	5	1.00		
One	178	24	4	1.12	(0.92–1.36)	*0.275*
Two or more	169	18	5	1.10	(0.90–1.35)	*0.370*
Missing data	261	8.5	4	1.68	(1.39–2.01)	*<0.001*

95% CI, 95% confidence interval; Mod Diff, moderately differentiated; Nephr, nephrectomy.

## Discussion

Our analysis reveals that CN is not being conducted in the majority of patients that present with synchronous metastatic RCC. There is a tremendous discordance between the population of advanced RCC enrolled in prospective interventional clinical trials in which 80–90% of the patients have had nephrectomy, and the application of these reported efficacy data to clinical practice in which the majority of patients do not undergo CN. There is a discrepancy between the results of retrospective population-based data such as the SEER cancer registry outcomes data and the IMDC registry data in which OS is significantly better with CN, and those seen in prospective randomized trial results such as the CARMENA and SURTIME studies that show that CN is unlikely to make an impact on improving outcomes in the setting of contemporary anti-VEGF therapy such as sunitinib (9). SEER data consistently demonstrate that lack of CN is the largest contributor of shorter OS in RCC patients with distant-stage RCC. The factors included within the IMDC are not captured within the SEER database, and hence, comparison of patients within these different registries is not possible. However, it is highly likely that the extent of the impact of each factor on OS may not be equal, and interaction between the factors is likely. In addition, it is possible that synchronous presentation of primary kidney tumor and metastases represents a distinct disease entity than the presentation with metastatic disease following a remote history of nephrectomy (>12 months) for localized renal cancer.

The advent of immune therapy with checkpoint inhibitors has introduced a new perspective to the sequencing of CN and systemic therapy in metastatic RCC. Preclinical data continue to emerge supporting the optimal use of immune checkpoint inhibition with the primary tumor in place, due to higher mutation load, increased heterogeneity of mutations and greater potential for anti-tumor CD8 T cells expansion and cytokine release (10, 11). These findings demand a deeper evaluation of the established paradigm of initial CN in metastatic RCC followed by systemic therapy. In fact, this standard is already being questioned in localized RCC by the current cooperative group trial EA8143/PROSPER, which randomizes patients to standard therapy of CN or neoadjuvant PD-1 inhibitor therapy with nivolumab followed by CN. In the metastatic disease also, deferral of nephrectomy as a potential method of enhancing the efficacy of immune checkpoint inhibition needs to be prospectively evaluated. SEER-18 limited use data do not include systemic agents, and this was a limitation of our analysis. A Southwest Oncology Group trial, S1931, is proposing to evaluate immune checkpoint inhibitor-based regimen combination with or without the addition of CN. The timing of surgery in the nephrectomy arm will be at the end of 12 weeks of immune therapy to optimize the impact of systemic therapy. The study design is that of a randomized phase III with overall survival as the primary endpoint.

## Conclusions

National population registry represented by the SEER database shows that the majority of patients (63%) with metastatic RCC do not undergo CN. Nephrectomy still represents a significant factor that predicts for improved OS outcome in advanced RCC within population databases such as SEER and NCDB. Median OS of 3 months in the non-CN group and that of 18 months for cases with CN suggests the large magnitude of the disparity. Patient demographics and tumor characteristics make a significant impact on the incidence of CN. The impact of comorbidities (number and type) appeared to be modest within the population represented in the Metropolitan Detroit SEER data and was not statistically significant. The optimal management strategy of patients with advanced RCC unable to undergo nephrectomy needs to be prospectively evaluated in future clinical trials, in the setting of contemporary systemic therapy.

The subgroup of synchronous primary and metastatic kidney cancer has been underrepresented in clinical trials. Future studies geared toward addressing the issues of optimal efficacy of therapy within this patient population are being planned. Patient and disease characteristics that determine decision of CN warrant further study and may represent a key to the enhancement of outcomes in advanced renal cancer.

## Conflict of Interest

The authors declare that they have no conflict of interest.
